# Groundwater Remediation of Volatile Organic Compounds Using Nanofiltration and Reverse Osmosis Membranes—A Field Study

**DOI:** 10.3390/membranes11010061

**Published:** 2021-01-16

**Authors:** Thomas J. Ainscough, Darren L. Oatley-Radcliffe, Andrew R. Barron

**Affiliations:** 1 Energy Safety Research Institute (ESRI), Bay Campus, Swansea University, Fabian Way, Swansea SA1 8EN, UK; T.J.Ainscough@Swansea.ac.uk (T.J.A.); A.R.Barron@Swansea.ac.uk (A.R.B.); 2 Centre for Water Advanced Technologies and Environmental Research (CWATER), College of Engineering, Bay Campus, Swansea University, Fabian Way, Swansea SA1 8EN, UK; 3 Department of Chemistry and Department of Materials Science and Nanoengineering, Rice University, Houston, TX 77005, USA; 4 Faculty of Engineering, Universiti Teknologi Brunei, Jalan Tungku Link, Gadong BE1410, Brunei

**Keywords:** groundwater, reclamation, nanofiltration, VOC

## Abstract

Groundwater contamination by chlorinated hydrocarbons represents a particularly difficult separation to achieve and very little is published on the subject. In this paper, we explore the potential for the removal of chlorinated volatile and non-volatile organics from a site in Bedfordshire UK. The compounds of interest include trichloroethylene (TCE), tetrachloroethylene (PCE), cis-1,2-dichloroethylene (DCE), 2,2-dichloropropane (DCP) and vinyl chloride (VC). The separations were first tested in the laboratory. Microfiltration membranes were of no use in this separation. Nanofiltration membranes performed well and rejections of 70–93% were observed for synthetic solutions and up to 100% for real groundwater samples. Site trials were limited by space and power availability, which resulted in a maximum operating pressure of only 3 bar. Under these conditions, the nanofiltration membrane removed organic materials, but failed to remove VOCs to any significant extent. Initial results with a reverse osmosis membrane were positive, with 93% removal of the VOCs. However, subsequent samples taken demonstrated little removal. Several hypotheses were presented to explain this behavior and the most likely cause of the issue was fouling leading to adsorption of the VOCs onto the membrane and allowing passage through the membrane matrix.

## 1. Introduction

The remediation of contaminated groundwater is a costly and complex process typically involving multiple stages and systems [1]. Water treatment plants vary greatly depending on the compounds and contaminants in the water source that need to be reduced or removed to below trace levels to meet local environmental standards [2]. A treatment plant can use a combination of physical, chemical and biological steps. A standard setup for hydrocarbon (including volatiles) remediation could include an oil–water separator (OWS) as an initial physical separation stage followed by an activated biological sludge and chemical dosing stage to remove oil emulsions below 150 microns and dissolved organics [3,4]. Often an adsorption stage is included and the selection of the adsorbent material is critical to success [5]. Other combinations of technology for VOC removal are also available and these may include the use of plasma, adsorption and catalysis [6] to name but a few.

Integrated membrane systems for desalination used in drinking water production typically combine a microfiltration (MF) or ultrafiltration (UF) membrane as a prefilter to preserve the integrity and avoid particulate damage of the reverse osmosis (RO) membrane by removing suspended solids and microorganisms [7,8]. Nanofiltration (NF) also plays a predominant role in the drinking water industry [9,10]—NF membranes were initially deployed as water softeners due to their unique capabilities of screening divalent and multivalent ions such as calcium (Ca^2+^) and magnesium (Mg^2+^) found in hard water areas [11]. Advances in the development of NF membranes has given NF membranes additional usages in water purification, specifically the removal of naturally occurring organic material including viruses and pesticides [12,13]. NFs are not capable of removing all organic material [14]. However, their inclusion is warranted to reduce fouling of the RO membranes by the organic material they can filter [15,16]. RO membranes will exclude all remaining divalent and multivalent ions, RO will also remove monovalent ions such as sodium (Na^+^) salts. One of the major costs in the remediation of a contaminated water source is the use of activated carbon adsorption removal of volatile organic compounds (VOCs) [17]. The occurrence of VOCs within local water sources is highly regulated due to the associated health concerns [18,19,20]. Due to the prohibitively expensive nature of activated carbon, the used cartridges can be regenerated using solvent regeneration [21,22], steam regeneration [23,24] or more commonly used thermal regeneration [25,26,27]. Thermal regeneration is conducted by pyrolysis, burning off adsorbed VOCs along with a carrier gas, mainly nitrogen, to remove the VOCs and regenerate the activated carbon. There are two major drawbacks with thermal regeneration—it requires considerable investment in a suitable furnace which will only become financially viable with a high enough quantity of activated carbon [28], and it causes carbon losses of 5–15 wt% [29] or a reduction in adsorptive capacity [30], the addition of new material to make up the absorptive losses is required. Thus, small to medium wastewater treatment plants will dispose of the spent activated carbon through an external company where it will be recycled or regenerated for a cost and a discount on fresh material that would be required is applied.

In this work, our research group was approached by a large multinational company, which will be referred to as Company A for confidentiality purposes, to investigate the use of membrane technology for the removal of VOCs to reduce the expenditure of activated carbon on Company A’s water treatment plants. Previous work [31,32] suggested that a novel super-hydrophilic ceramic microfiltration membrane could be used for this purpose. The study also investigated the use of nanofiltration and reverse osmosis membranes for the application which was conducted using laboratory trials and on site technology deployment.

## 2. Experimental

### 2.1. Background Information and Preliminary Testing

The remediation process at the Bedfordshire site involves the clean up of contaminated groundwater and the treatment process involves both liquid and vapor VOC abatement processes; both of which contain granular activated carbon as a treatment process. The groundwater from specifically dug wells in high-contamination areas is pumped to an OWS. The decanted water is passed through an air stripper to remove pollutants with high Henry’s Law coefficients such as trichloroethylene (TCE) and tetrachloroethylene (PCE). The air-stripped VOCs are directed to the vapor treatment process, and the groundwater with any remaining dissolved VOCs is passed through a biological treatment step followed by the granular activated carbon adsorption stage. There is a chemical additive stage prior to being reinjected back into the ground wells.

The contaminated water source is an historically polluted source from a redundant production process, and the water contained over 10 VOCs above trace GC–MS detection. The EPA environmental limit for the compounds found in the water source is 5 ppb, and five compounds were regularly detected above this level—they were TCE, PCE cis-1,2-dichloroethylene (DCE), 2,2-dichloropropane (DCP) and vinyl chloride (VC). The volatile organic compounds had molecular weights between 62.50 g mol^−1^ (VC) and 165.83 g mol^−1^ (PCE). The intrinsic hydrophobicity of these compounds varied significantly, as was reflected by the logarithm of their octanol–water partitioning coefficient (XLogP3). As can be seen in Table 1, the properties of the selected volatile organic compounds demonstrated that some compounds are hydrophilic (XLogP3 > 2.5) while others are hydrophobic (XLogP3 < 2.5) and ranged between 1.5 and 3.4. The groundwater at the Bedfordshire site has very little organic content compared to Company A’s other global sites. However, the global scope of the project covers any contaminated groundwater source Company A may have. The project initiated between Swansea University and Company A was to develop a system of removing organic matter contamination from groundwaters on various sites with an initial test site located in the UK. The methodology used in this study was to be based upon the development of a novel super-hydrophilic ceramic membrane system as previously reported [31,32]. Preliminary investigations from this project increased operational understanding of the separation mechanisms of the modified ceramic membrane and have shown that the membrane is very good for separations where the contamination levels are very high. In heavily contaminated systems, there is in effect a biphasic mixture of organic and aqueous components (with some low-level organics dissolved in the aqueous phase). The modified ceramic membrane has been shown to be extremely effective at separating these two phases. However, the low-level organics dissolved within the aqueous phase are not separated to a high degree in this process. Thus, to produce an ultra-clean aqueous stream, an additional NF or RO process is proposed as a polishing step. For this reason, the site strategy for deployment of a pilot system at the site was modified to use a polymeric NF and/or RO membrane.

#### 2.1.1. Pilot-Scale Testing of Ceramic Microfiltration Membranes

**Figure 1:**
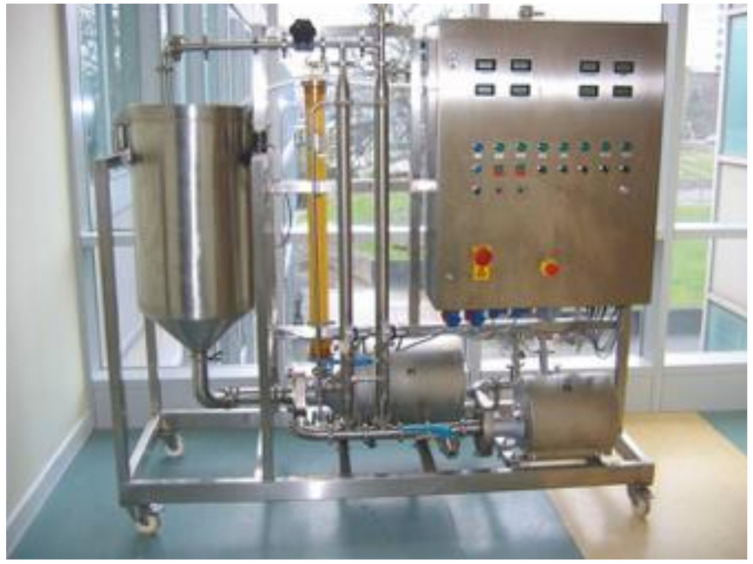
Swansea University ceramic pilot microfiltration membrane rig.

All preliminary ceramic membrane filtrations were conducted on a Swansea University MF/UF pilot-scale membrane rig, as pictured in Figure 1. The system used is overengineered for the application required. However, the use of two pumps allows the pressure and cross-flow velocity to be independently controlled, which is beneficial in a research environment. The system was originally designed to be operated as both a micro and ultrafiltration system, capable of operating at pressures of up to 6 bar. However, the system can operate at the much lower pressures required for microfiltration using inverters for the pumps. The system consists of a 100 L stainless steel feed tank, which feeds into the feed pump (Fristam FPE 742, Fristam Pumpen KG, Hamburg, Germany) which can deliver up to 5.5 bar pressure at a flow rate of up to 10 m^3^/h. The flow then enters the second pump (P2: Fristam FPE 722, Fristam Pumpen KG, Hamburg, Germany), which is capable of delivering up to 3.8 bar at a flow rate of up to 10 m^3^/h. The flow from P2 then passes through the membrane module. The retentate flows out of the membrane module and enters the optional heat exchanger system. The heat exchanger consists of two shell and tube heat exchangers connected to a cold water and/or steam supply as required. Cold water supply is controlled by a solenoid (Burkert 6213 A, Burkert, Ingelfingen, Germany) connected to a thermostat on the control board. The retentate re-circulates around the loop and returns to the feed tank via a diaphragm valve which sets the loop pressure (large black valve at the top of Figure 1). The system was operated at several pressures controlled via the pumps (P1 and P2) and the return valve. A temperature of 25 °C was maintained throughout the experimental work controlled by the solenoid valves attached to the heat exchangers controlling the steam and water flow rates.

An industrial-scale Pall Membralox 0.2 µm ceramic microfiltration membrane was used (Pall, Portsmouth, UK). The membrane active layer and support are made from alpha-alumina, α-Al_2_O_3_, and with an active area of 0.24 m^2^, the membrane is capable of handling temperatures of up to 95 °C, with a full pH range of 0–14 and a maximum pressure of 8 bar.

#### 2.1.2. Small-Scale Laboratory Testing Equipment for Nanofiltration and Reverse Osmosis

**Figure 2:**
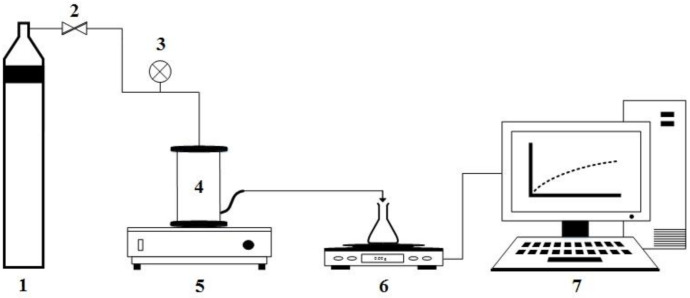
Frontal filtration experimental setup (1—nitrogen gas bottle, 2—pressure regulator, 3—pressure indicator, 4—Membranology HP350 stirred cell, 5—magnetic stirrer plate, 6—weight balance, 7—computer data logger).

All preliminary NF experiments were conducted at room temperature (22 ± 1 °C) and pH 6.5 ± 0.2, which is the pH of the deionized water (DI) water used throughout the study (Millipore Elix 5, Watford, UK). The filtrations were carried out using a commercially available stirred frontal filtration system (Membranology HP350 Filtration Cell, Membranology Ltd., Swansea, UK), previously described by Oatley-Radcliffe et al. [33] and illustrated in Figure 2. The cell has an operating capacity of 350 mL feed solution and an effective membrane surface area of 41.8 cm^2^. The filtration solutions were stirred magnetically at 300 rpm, the maximum practical stirrer speed previously determined [34].

Prior to first use, all membranes were soaked in DI water for 24 h. At the start of each series of experiments, the membrane was flushed with DI water at 30 bar for 1 h or 350 mL of filtrate, whichever was achieved first, to reach a steady compressed permeate flux. Following the compression experiment, a membrane clean water flux was recorded for 5, 10, 15, 20, 25 and 30 bar. Rejection experiments were then conducted at the previously stated pressures using toluene at a concentration of 1 g L^−1^ and a sample of groundwater prefiltered through a 0.2 µm microfilter to remove sediment. The concentrations of toluene and groundwater for the feed solution and permeate samples were analyzed using a total organic carbon analyzer (Shimadzu TOC-LCPH, Shimadzu Corporation, Milton Keynes, UK). Rejection measurements were based on 20 mL of permeate once the initial 5 mL of permeate was discarded, with 25 mL removed in total. After each rejection experiment the membrane was rinsed with DI water to remove any residual materials. Following a period of testing, the membrane pure water flux was retested to assess any deterioration in the membrane performance; this is a simple check for any potential fouling.

### 2.2. Nanofiltration/Reverse Osmosis Pilot for Deployment at the Bedfordshire Site

**Figure 3:**
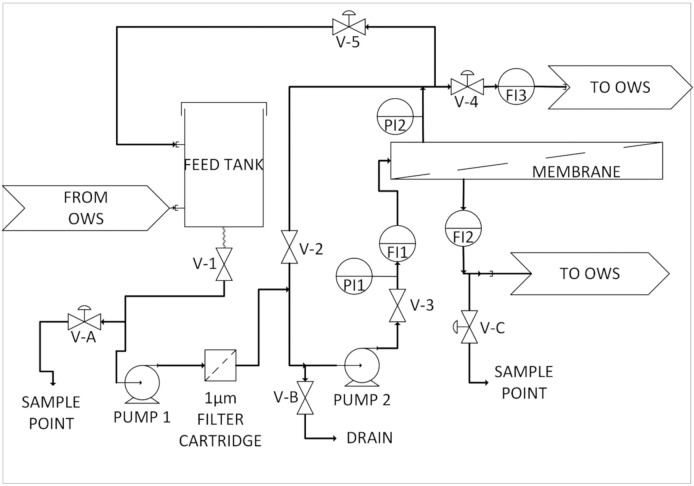
A schematic drawing of the pilot membrane rig used for the Bedfordshire site trials.

**Figure 4:**
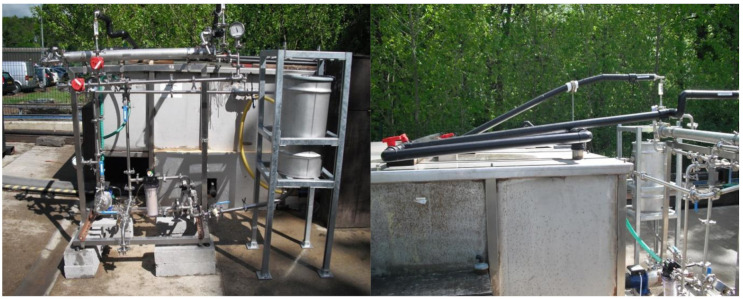
Pilot membrane rig used for the Bedfordshire site trials. Left: front view of the pilot. Right: connection to the existing Bedfordshire OWS unit.

The Bedfordshire site trial was conducted over a 4 month period, with an initial setup and commissioning exercise lasting two days. Due to the limited capacity of the electrical supply identified at the water processing unit, the deployment of a full-scale pilot system was not possible. A simplified pilot rig was constructed to avoid overloading the electrical capacity at the site; however, this simplified rig was not capable of the full operational range normally expected for a membrane process. Most notably, the applied operating feed pressure of this simple rig was limited to only 3.0 bar, with a maximum deliverable pressure of 3.5 bar. The rig was capable of operating in several modes using both polymeric NF and RO 2.5″ membrane modules separately. The membrane system, illustrated in Figure 3, consisted of a custom fabricated stainless steel feed tank (Axium Process Ltd., Hendy, UK) and in house-built Unistrut frame designed to be fully adjustable to allow the tank to be gravity fed from the OWS whilst also preventing the OWS from being fully drained. From the feed tank, pump 1 (Lowara-4HMS3/A) was used to provide pressure and top up flow to the membrane system. Following the pump was a filter cartridge housing (Pentek-3G housing) containing a string wound polypropylene 1.0 micron pre filter (Prosep) used to protect the NF or RO membrane from particulate debris. A second pump (Lowara-4HMS3/A) was used to provide circulation flow through the NF or RO membrane. After the second pump, a pressure gauge (Wika 232.50 and L990.22 Sanitary Seal) indicated the pressure of the membrane feed. A paddle flowmeter (Burkert S030 DN25 and Burkert 8035) allowed the flow rate through the membrane to be recorded, a parameter needed for calculating the cross-flow velocity needed for understanding the membrane performance throughout the trial. Both membranes were contained within a custom stainless steel housing (Axium Process Ltd., Hendy, UK). A second pressure gauge after the membrane indicated the pressure of the membrane retentate, in conjunction with the first pressure gauge the transmembrane pressure can be calculated to allow the calculation of the membrane flux per bar across the membrane. A flowmeter (Omega Engineering) was placed on the membrane retentate return to the OWS. This flowmeter was switched out between a FL7205 and FL2098 depending on operation of the rig, with the majority of retentate being recycled to the Swansea feed tank or back to the OWS, respectively. In addition, a flowmeter (Omega Engineering-FL7201) on the membrane permeate outlet was used to monitor the permeate flow rate, a parameter used to monitor the membrane performance by calculating the membrane flux using the known surface area of the membrane. A schematic of the pilot plant is included in Figure 3 and photographed in Figure 4.

All materials processed during the site trial were removed from the existing OWS within the groundwater facility and returned to the OWS following processing, i.e., there was no risk of non-treatment leading to non-compliance of the site during the trial period.

Throughout the trial, either total organic carbon (TOC) analysis conducted by Swansea University (Shimadzu, TOC-LPH, Milton Keynes, UK) or gas chromatography mass spectrometry (GC–MS) conducted by Natural Resources Wales (NRW, Llanelli, UK) using a method equivalent to that of the EPA 8260b for volatile organic compounds (VOCs) was used to analyze the separation performance.

The NF membrane used was a GE Osmonics DK series (model: DK2540F1073). The RO membrane used was a GE Osmonics AK series (model: AK2540TM).

### 2.3. Laboratory Analysis

#### 2.3.1. General Laboratory Filtration Trials

Salt rejection was measured at the previously stated pressures using sodium chloride (NaCl) (Fisher Scientific, Loughborough, UK) at a concentration of 2000 ppm, and the conductivity of feed and permeate samples was measured using a conductivity probe (Jenway Model 3450). Toluene rejection was measured at the previously stated pressures using toluene (Reagent grade, Fisher Scientific, Loughborough, UK) at a concentration of 100 ppm, and the concentrations of feed and permeate samples were measured using a total organic carbon analyzer (Shimadzu TOC-LCPH, Shimadzu Corporation, Milton Keynes, UK). TCE rejection was measured on its own at the previously stated pressures using TCE (Puriss ≥ 99.5% (GC), Sigma, Gillingham, UK) at a concentration of 100 ppm, and the concentrations of feed and permeate samples were measured using a total organic carbon analyzer (Shimadzu TOC-LCPH, Shimadzu Corporation, Milton Keynes, UK). A feed VOC mixture of TCE, PCE, DCE and DCP (Sigma, Gillingham, UK) was created with a concentration of 100 ppm for all solvents. For all feed batches, the VOCs were initially dissolved in 50 mL of methanol (Fisher Scientific, Loughborough, UK) and added to a 5 L flask. A further 50 mL of methanol was added to the measuring cylinder and transferred to the 5 L flask to avoid any residual VOCs clinging to the glassware. VOC rejection was measured at the previously stated pressures. Feed and permeate samples were measured using a headspace GC–MS system at Swansea University (Agilent GC 6850, MS 5977A, HS 7697A 12-vial) not previously available during the preliminary and pilot-scale trial. Two calibrations were conducted—a ppb calibration and ppm calibration—with the following standards: blank, 10, 50, 100, 250, 500, and 1000 ppb and 1, 10, 50, 100, and 150 ppm, respectively. Data acquisition and analyses were performed using the MassHunter Workstation software with quantification using the selected ion monitoring (SIM) method. See Table 2 for GC–MS HS method.

All rejection measurements analyzed by TOC were based on 20 mL of permeate once the initial 5 mL of permeate was discarded, with 25 mL removed in total. All GC–MS rejection measurements were based on 10 mL of permeate once the initial 5 mL of permeate was discarded, with 15 mL removed in total. After each rejection experiment, the membrane was rinsed with DI water to remove any residual solvent. Following each experiment set—NaCl, toluene, TCE and VOC mixture, respectively—a clean water flux was conducted at 20 bar to ensure the membrane was not deteriorating or fouling.

#### 2.3.2. Contact Angle Measurements

Contact angles of the dry membranes were measured using a VCA Optima contact angle analyzer (AST Products Inc., Billerica, USA) using the static sessile drop method. A droplet of ultrapure water obtained from the Millipore Elix 5 was delivered onto the dry membrane surface and a static image of the droplet was taken after contact with the surface. Contact angle measurements performed using the VCA Optima software for each membrane at 5 different locations were recorded and the average taken. Contact angles and standard deviations are included in Table 3a,b.

#### 2.3.3. Membranes Used

The membranes used in this study were obtained in either flat sheet format for laboratory trials or spiral wound format for pilot and deployment trials. Four NF membranes were used in total; namely, the DK and DL membranes sourced from GE Osmonics and the NF90 and NF270 sourced from Dow Filmtec. Five RO membranes were used and these were the AK and AG (GE Osmonics) and the BW30, BW30LE and BW30XFR (Dow Filmtec). Full details of the NF and RO membranes are illustrated in Table 3a,b, respectively.

### 2.4. Rejection Theory

The experimental rejection characteristics of a membrane are usually defined by the observed rejection:

(1) $$R_{\mathrm{obs}}=1-\frac{C_{P}}{C_{F}}$$

where C_F_ and C_P_ are the concentrations of the feed and permeate, respectively. However, due to concentration polarization, the concentration at the membrane surface, C_W_ is higher than that of the bulk feed concentration, C_F_. Therefore, real rejection of the solute, R_real_, which is always equal to or greater than R_obs_ is defined as:

(2) $$R_{\mathrm{real}}=1-\frac{C_{P}}{C_{W}}$$

The concentration at the wall, C_W_, can be calculated indirectly using a suitable model for concentration polarization [33,34,35]. The approach to concentration polarization taken in this study is that of the infinite rejection method first reported by Nakao and Kimura [36] and given as:

(3) $$\exp\left(\frac{J_{v}}{k}\right)=\;\frac{C_{W}-C_{P}}{C_{F}-C_{P}}$$

where k is the mass transfer, defined as:

(4) $$k=\;\frac{D_{\mathrm{eff},\infty }}{\delta }$$

and $D_{\mathrm{eff},\infty }$ is the diffusion coefficient at infinite dilution and δ is the thickness of the concentration polarization layer.

The mass transfer coefficient may be determined experimentally by the substitution of Equations (1) and (2) into Equation (3), yielding:

(5) $$\ln\left(\frac{1-{\;R}_{\mathrm{obs}}}{R_{\mathrm{obs}}}\right)=\;\frac{J_{v}}{k}+\;\ln\left(\frac{1-{\;R}_{\mathrm{real}}}{R_{\mathrm{real}}}\right)$$

In this case, the mass transfer coefficient may be represented as

(6) $$k={a\omega }^{n}$$

where a and n are predetermined constants and ω is the stirrer speed. For the Membranology cell, these constants are 2.993 × 10^−6^ and 0.415, respectively [34].

## 3. Results and Discussion

### 3.1. Preliminary Experiments

As previously alluded to, our understanding of the modified ceramic separation capabilities changed during the preliminary investigations into their use for this study. Previous and ongoing work using frack-produced water has demonstrated that the membranes are capable of cleaning water at the molecular level [32]. These waters are heavily contaminated far beyond solubility limits, resulting in biphasic mixtures and oil–water emulsions. Under these conditions, the modified ceramics are capable of separating small hydrocarbons such as toluene. However, upon attempting to use these membranes on a saturated toluene solution in just DI water, 1 g L^−1^ at 20 °C, no separation occurred. Therefore, the modified ceramics would not be suitable for the Bedfordshire trial, or at least not the only type of filter, hence exploratory NF laboratory studies were conducted to assess potential. The same toluene–water concentration solution that was showing 0% rejection with the ceramic membranes was rejecting between 70% and 93% of toluene using a NF membrane with a transmembrane pressure (TMP) of 2.5–20 bar, respectively. See Table 4.

With a successful separation, a further experiment using a 350 mL sample of the Bedfordshire site water was run. The sample was prefiltered by a 0.2 micron ceramic membrane prior to feed analysis and NF filtration occurring. It was first discovered during this experiment that the total organic carbon content of the feed water was considerably lower than expected. The results of the test are shown below in Table 5.

The NF DK series showed excellent overall organic carbon removal even at relatively low pressures. Unfortunately, the GC–MS equipment was not available at this stage to test specific volatile organic removal capabilities of the membrane.

### 3.2. Pilot Scale

#### 3.2.1. Nanofiltration

**Figure 5:**
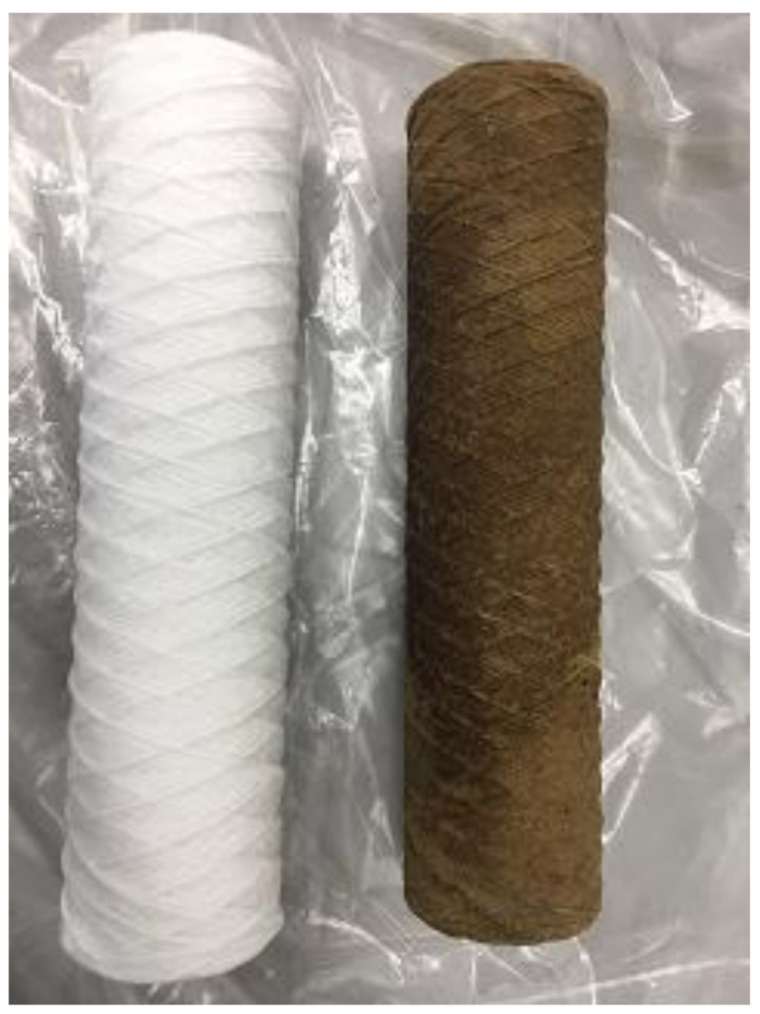
An example of a virgin prefilter (**left**) and a fouled prefilter (**right**).

The initial setup employed a GE DK series NF membrane, set to operate with most the retentate (concentrated dirty water) from the membrane directed back to the Swansea feed tank, whilst a small bleed of retentate and clean water permeate was returned to the OWS. In this configuration, if the membrane performed well and separated the volatile organic carbons, then the concentration of these organics in the Swansea feed tank would steadily increase. Further, the configuration removes only a small quantity of liquids from the OWS to replenish the lost permeate from the pilot rig to avoid increased mixing within the OWS. After 4 weeks of operation, the configuration was changed to return both the retentate and the permeate back to the OWS. In this mode, the membrane process is no longer a recycle of retentate or concentration process, but a once-through continuous process more suitable for larger-scale operations. During the once-through trial period, an observation was made that the prefilter experienced considerable fouling compared to the recycle mode of operation. See Figure 5. This was most likely the result of the increased flow of liquid from the OWS causing settled sediment within the OWS to be dispersed and transferred to the pilot system. There is also the possibility that during this period of operation, the well pumps were sending more silt to the OWS. Prior to deployment, it was recommended that a prefilter be installed in the system as a precaution despite the OWS water containing ‘minimal solids.’ Throughout the trial, the solids concentration of the OWS water was above what would be considered minimal for a NF membrane system under normal operation—a permanent system would have to cope with this level of solids more appropriately than weekly filter changes such as the incorporation of the functionalized ceramic microfiltration membrane. The decision was made to return to the original configuration to prevent starving the second pump or deadheading the first pump should the prefilter block completely.

**Figure 6:**
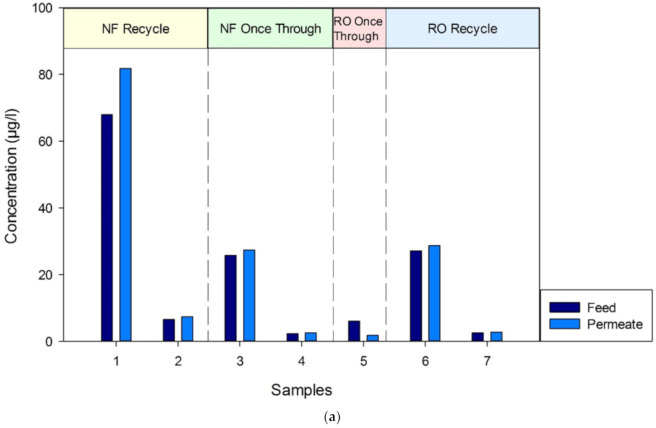

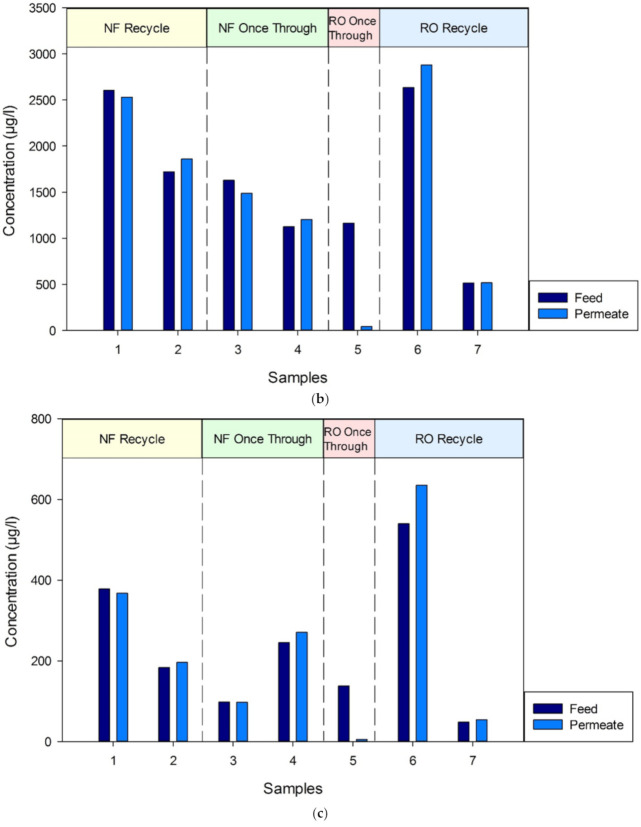

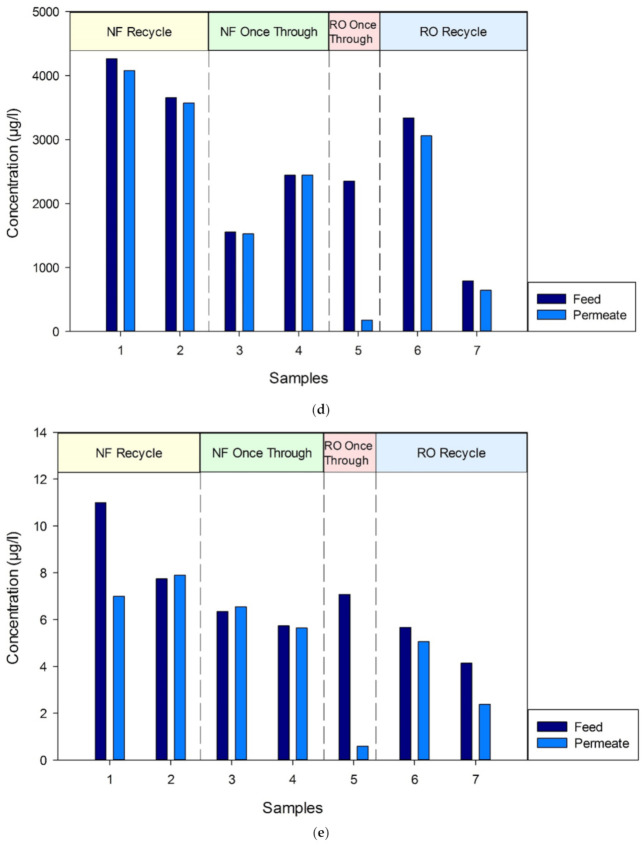
(**a**) Graphical representation of GC–MS data for vinyl chloride throughout the pilot trial. (**b**) Graphical representation of GC–MS data for cis-1,2-dichlorothylene throughout the pilot trial. (**c**) Graphical representation of GC–MS data for 2,2-dichloropropane throughout the pilot trial. (**d**) Graphical representation of GC–MS data for trichloroethylene throughout the pilot trial. (**e**) Graphical representation of GC–MS data for tetrachloroethylene throughout the pilot trial.

The feed to the pilot from the Bedfordshire OWS contained five major compounds in varying quantities, totaling between 7358 and 1360 ppb across the trial period. The GC–MS data of the five main compounds are shown graphically in Figure 6a–e for VC, DCE, DCP, TCE and PCE, respectively.

For both operating configurations, the GC–MS method for VOCs detected very little or no separation occurring during the NF trial, with the feed and permeate samples being comparable. As an example, sample data from 27th May of the five main compounds, shown in Table 6, clearly show no separation of the VOCs occurring.

The results for the NF were disappointing but not completely unexpected if the membranes were to follow normal separation principles. It is well known from literature [37] that rejection of a solute is dependent on the applied pressure for steric rejection—the higher the applied pressure, the higher the rejection. At low pressures, or Bedfordshire trial operating pressures, the rejections are considerably lower than the rejection expected at typical operating pressures of NF and RO membranes. During the NF stage of the trial, TOC analysis was conducted in conjunction with the GC–MS analysis. As shown in Table 7, on average, 75% of the total organic carbon was removed by the NF membrane.

A rejection of 75% organic carbon is a much more positive result than the equivalent VOC rejection—the 1 µm prefilter (microfilter) will absorb only a fraction, if any, dissolved organic carbon. This is clear evidence that the NF membrane is still separating some organics, if not quite as efficiently as the laboratory conditions—the use of an NF is not entirely redundant as the removal of organics will assist in preventing an RO membrane downstream from being fouled. When trying to understand why the membrane displayed reduced organic carbon, the harsher conditions found in real-world trial conditions and the damage potentially inflicted on the membrane module must be considered. If the membrane had been damaged or pore sizes enlarged by the water contaminants, then the permeate flux would have been much greater than a flux of 5 LMH/bar, as suggested by the manufacturer. Upon closer examination of the recorded permeate flow rates and operating pressures during the trial, the membrane flux performance changed very little throughout the trial, with the specific flux averaging 5.45 LMH/bar ± 0.5 during operation. See Table 8.

Therefore, the membrane was not inadvertently damaged by dissolved solids or other materials. Despite the nanofiltration membrane not removing the VOCs, it effectively removed a large percentage of other organic carbons in the water source. As previously noted, the operating pressure of 3 bar is very low for a NF membrane of this type. A minimum operating pressure would usually be 8 bar(g), with a preferred operating pressure upwards of 20–30 bar(g). Increasing the operating pressure could potentially improve the rejection characteristics of the membrane. However, the lack of rejection of the VOCs even at 3 bar(g) suggests that VOC removal may not be viable with an NF membrane and only a trial using more appropriate equipment could confirm this fact. However, the initial proposal of using a ceramic microfilter required a reduced pressure system. To confirm this conclusion further in-house laboratory testing was necessary to gather rejection data at higher pressures (5–30 bar).

**Figure 7:**
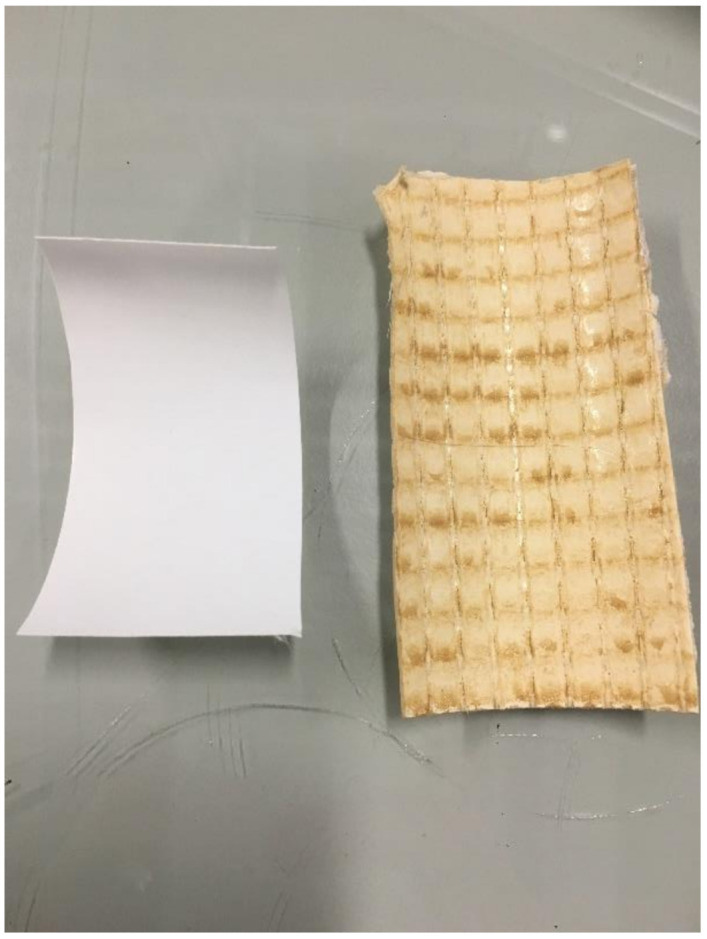
New DK membrane (**left**) vs. post trial DK membrane (**right**).

Upon analyzing the used pilot membrane, the membrane surface was considerably fouled see Figure 7. This fouling layer does not appear to have affected the permeate flow rate due to the pore size of the membrane being considerably smaller than the expected pore size of the foulant. However, this filter cake could have significantly affected the separation properties of the membrane, negating the surface charge effects [38,39].

#### 3.2.2. Reverse Osmosis

Prior to the Bedfordshire deployment, there were concerns that the nanofiltration membrane would not be fully effective at removing the VOCs to below the levels desired due to the pore size and reduced pressures being used. As a result, a low-energy RO membrane was purchased ready to switch out the nanofiltration membrane during the trial if necessary. A low-energy RO is designed to operate at a much lower pressure, 7 to 10 bar(g), than normal RO pressures (>60 bar) while separating 98% of NaCl versus 99.5% NaCl, respectively. Despite the Bedfordshire pilot rig pressure being limited to 3 bar, the low-energy RO membrane was anticipated to yield better separation than the NF membrane and considerably better separation than a standard RO membrane.

Due to circumstances outside of our control, TOC analysis was unavailable during the RO trial period. The GC–MS results for VOCs showed 92.8% removal on the first sample taken after an hour of operation. See Table 9.

This was a very promising result considering the membrane was being operated at a pressure considerably less than optimum, suggesting that a further increase in pressure would increase the separation capabilities beyond 93% rejection of the VOCs. However, the following sample (two weeks later) and those beyond showed no VOC separation. See Table 10.

Once again, if the membrane had been damaged, then the permeate flux would have been much greater than the initial flux when the membrane was first installed. The results indicate that the specific flux did not change significantly during the sample period. See Table 11.

**Figure 8:**
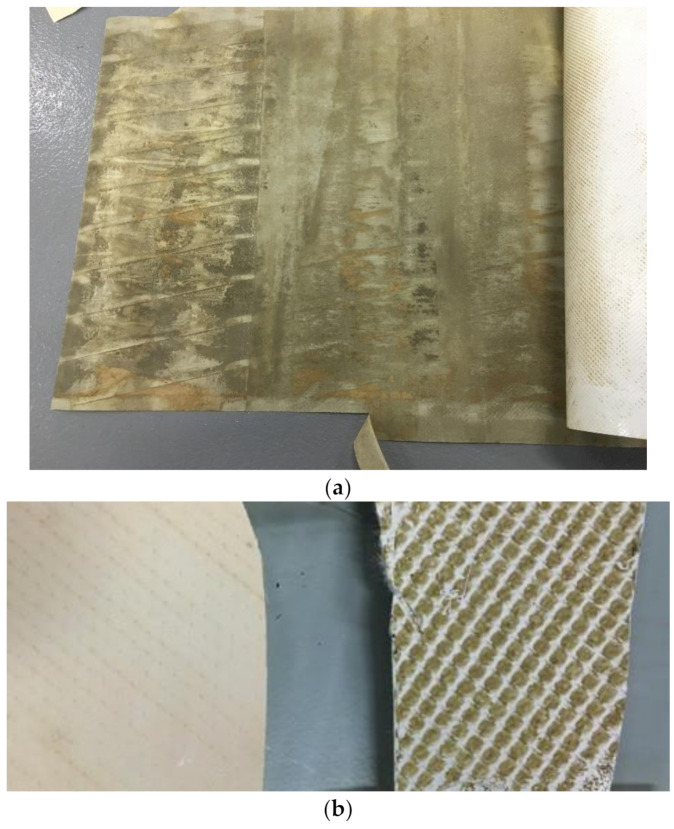
Illustration of the fouling that occurred for the RO membrane. (**a**) Fouled backing layer; (**b**) virgin membrane surface [left] vs. post trial membrane surface [right].

The membrane does appear to have experienced considerable fouling towards the end of the trial as the specific flux drops to approximately 29% of the original value. Membrane fouling can affect the quality of rejection, the surface charge of the membrane can be severely impacted by the build up of a fouling layer, and this may well explain the results seen [40,41]. Also of note during this period of operation, the prefilter experienced considerable fouling by silt. It is possible that the silt saturated the microfilter, and was forced through to the RO membrane and that this then had a detrimental effect on the membrane performance. This silt contamination was confirmed post trial. See Figure 8a,b. In addition, the dissolved organic material rejected by the NF membrane would also be rejected (and to a higher extent) by the RO membrane and could possibly cause fouling. However, without GC–MS data for the latter stages of the RO trial and no TOC data throughout, a categorical confirmation that fouling is the reason behind this reduced separation is not possible from the pilot-scale trials.

**Figure 9:**
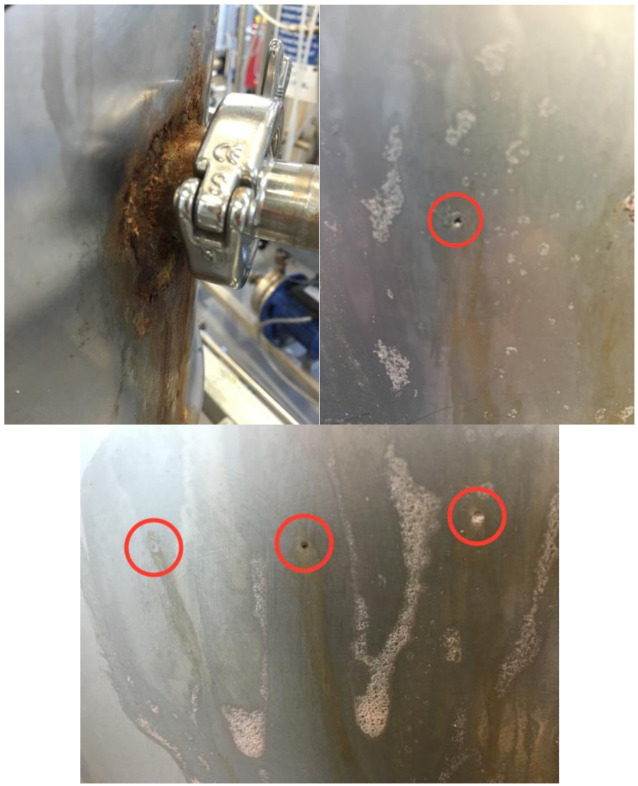
Swansea feed tank corrosion. Top left, rusting around the tri-clamp connection. Top right and bottom, pitting on the vessel surface.

As with the NF trial, further testing on the RO membranes across an increased pressure range, including TOC and GC–MS analysis at the Bedfordshire site conditions and the operating conditions suggested by the manufacturer, was required. At the same time, an investigation into the fouling effects on the membrane under more stringent monitoring conditions should be conducted. This will determine whether fouling caused the rejection of the VOCs to drop so drastically. As a final note on the pilot-scale trial, during decommissioning of the system, it was discovered that one of the side port ferrules had been severely rusted on the stainless steel feed tank. Further, multiple pin holes were found on the side of the tank. See Figure 9. The tank was returned to the manufacturer for diagnostic analysis. The manufacturer’s report indicated that the tank was made of an inferior quality stainless steel, 304, while all other components of the system were 316 L. The ferrule issue was also a result of the stainless steel used and the thickness of the tank. The ferrule was 316 L grade, and as a result, 316 L weld filler was used, which requires a higher temperature than 304 weld filler. When heated to the higher welding temperatures, the chromium combines with the carbon, leaving the steel short of chromium. Therefore, any further systems should be fully constructed from 316 L as a minimum. However, the use of a suitable plastic such as PVC or HDPE would be recommended to considerably reduce the cost of any subsequent system.

#### 3.2.3. Contact Angle

The contact angle was measured for the fouled DK and AK pilot-scale trial membranes, and the DK NF membrane hydrophobicity increased significantly to 72.88 ± 0.78°. The AK RO membrane, on the other hand, behaved in the opposite manner—the hydrophilicity increased to 48.76 ± 2.52°. Similar phenomena have been reported previously [39,42].

### 3.3. Laboratory Scale

#### 3.3.1. TOC Analysis

Additional testing under laboratory conditions was necessary to understand the results of the pilot-scale trial. As well as the GE DK membrane used in the pilot scale, another three NF membranes were tested, as listed in Table 3a. As for RO, together with the pilot-scale RO membrane, GE Osmonics AK, an additional four RO membranes were assessed, as shown in Table 3b. The first experiment was investigating salt rejection, and the reason for this test was twofold. Firstly, membrane manufacturers supply NaCl rejection data for RO at a specific operating condition, and therefore this testing allows comparison with the manufacturer’s published results as a secondary check that the membrane is operating as expected. Secondly, the treated groundwater will eventually be released back into the local water supply, and since a change in groundwater salinity may have an adverse effect on the local environment, this must be monitored and balanced. The second set of experiments using toluene, an immiscible aromatic hydrocarbon, were used to replicate a very common groundwater contamination of a water–oil biphasic mixture with extremely low solubility, 0.52 g/L at 20 °C. The final set of experiments were on a TCE–water mixture, the predominant contaminate found in the groundwater at the Bedfordshire site. TOC analysis was used to analyze the rejection as the GC–MS was initially unavailable. Results from all three experimental sets conducted for nine membranes can be found in Table 12a–i.

The rejection of NaCl for NF membranes is not widely published by the manufacturer as NF is primarily used as a water softener, typically removing divalent salts not monovalent. However, literature values suggest that a NaCl rejection of 5–95% can be expected for NF depending on feed salinity and operating conditions [43]. Clean water fluxes obtained from the new membranes confirmed that the membranes were operating effectively. Toluene is a relatively inexpensive solvent, making it ideal as an initial solvent to test the performance of the NF membranes. As previously discussed, toluene has a low solubility with water at room temperature. When the concentration exceeds the solubility limit, the solution forms a biphasic mixture with a top layer of toluene. Due to this nature of biphasic mixtures, representative sampling can be particularly difficult to achieve. To avoid these difficulties, the feed concentration selected, 0.1 g L^−1^, removes the potential for inaccurate feed concentration samples. For all four NF membranes, the observed rejection decreases as the applied pressure increases. For example, for Dow NF90, the observed rejection is 90.4% to 51.7%, between 5 and 30 bar, respectively—this is counter to the real rejection, which takes concentration polarization at the membrane surface into account. The real rejection ranges between 88.1% and 99%, respectively, and the greatest difference between the observed and real rejection, ≥60%, is at the highest pressures. This occurrence is sensible, since as the pressure increases, the flux rate increases, and the resulting mass transfer is governed by convective transport of solute to the membrane surface. This convective flux to the membrane surface is significantly higher than the mixing rate removing solute from the membrane surface and thus concentration polarization is inevitable. The rejection data obtained for TCE demonstrate a similar pattern. However, the observed and real rejections are both lower when compared to the equivalent toluene results. If nanofiltration separation was based solely on steric (size) exclusion, then we would expect to see toluene rejecting less than TCE due to its lower MW. However, previous studies have proven that NF exhibits a combination of separation mechanisms from both UF and RO, steric and Donnan (charge) exclusion or ionic diffusion, respectively [44,45,46]. All four membranes exhibit a similar decline in observed rejection from 5 to 15 bar, to varying degrees. Between 15 and 30 bar, the rejection appears to reach a plateau, with only some slight fluctuations observed.

The rejection of NaCl for RO membranes is published by the manufacturer, allowing a cheap and relatively simple way of testing the membranes integrity. GE Osmonics market the AK as a low-energy brackish water reverse osmosis (BWRO) with a minimum NaCl rejection of 98% using a 500 ppm NaCl solution, with operating conditions of 8 bar pressure, 25 °C and pH 7.5. As shown in Table 12e, the observed rejection was less than the advertised minimum. However, this can be explained as the feed solution used was 4-fold above the manufacturer test solution, 2000 ppm. A higher feed NaCl concentration increases the salt gradient between the feed and permeate or osmotic difference. An increase in osmotic gradient requires a higher applied pressure to overcome the osmotic pressure to maintain the permeate flux. With a constant pressure, the water flux decreases, and therefore salt passage increases. GE Osmonics tests on the AG series use a 2000 ppm NaCl solution, with operating conditions set as 15.5 bar operating pressure, 25 °C and pH 7.5. The AG series is marketed as a standard BWRO with a minimum NaCl rejection of 99%. It is unclear why GE use two different feed solutions for comparable membranes. As shown in Table 12f, the observed rejection was 99.2% at 15 bar. Dow Filmtec publicize the BW30 as a low-energy BWRO with a minimum NaCl rejection of 99% using a 2000 ppm NaCl solution, with operating conditions of 15.5 bar pressure, 25 °C and pH 8. As shown in Table 12g, BW30 only obtained a salt rejection of 96.1%. BW30LE, a low-energy BWRO, has a minimum NaCl rejection of 98% using a 2000 ppm NaCl solution, with operating conditions of 10 bar pressure, 25 °C and pH 8. As shown in Table 12h, BW30LE only obtained an observed salt rejection of 94.3%. The final Dow Filmtec membrane, BW30XFR, is an optimized extra fouling-resistant BWRO membrane. Dow claim a minimum salt rejection of 99.4%, and operational conditions are the same as those used in BW30. When tested, the membrane attained a salt rejection of 96.8%, as noted in Table 12i. It is unclear whether the manufacturers’ stated minimum rejections are observed or calculated real rejections. It is assumed that the published data are observed, but if the results are real, the results in this study are closer to the manufacturers’ values. The RO membranes exhibit the same separation tendencies as the NF membranes for toluene and TCE, but with higher observed rejections for all the RO membranes. As reverse osmosis membranes have no physical pores, size exclusion is no longer a separation mechanism. Solute passage is determined purely by solution diffusion, thus, the increased rejection for the RO membranes is entirely logical. Comparing the observed rejection and calculated real rejection, the variances between them are less pronounced compared to the NF membranes. This fully agrees with the NF filtration pressure increase vs. observed/real rejection theory—increased convective flux results in increased concentration polarization. RO membranes have a greatly reduced specific flux when evaluated against NF membranes. A lower permeate flux produces less convection to the membrane surface, which in turn minimizes concentration polarization, hence, less discrepancy between observed and real rejection.

#### 3.3.2. GC–MS Analysis

**Figure 10:**
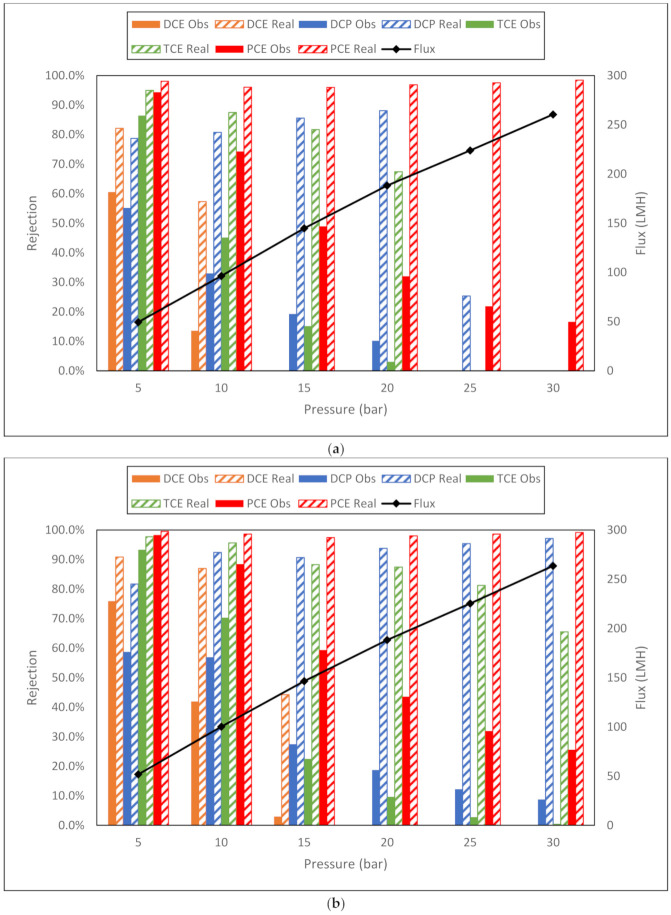

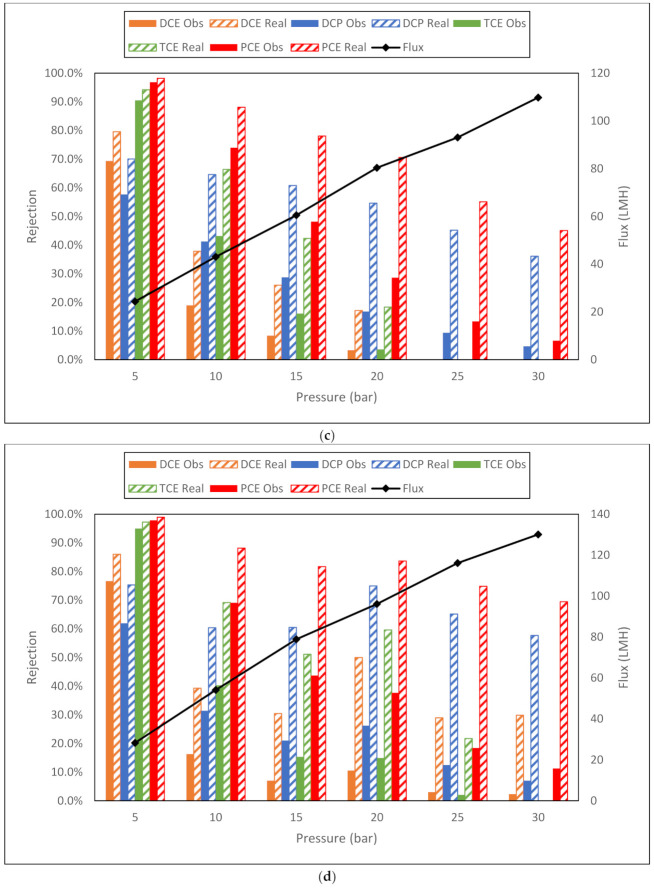

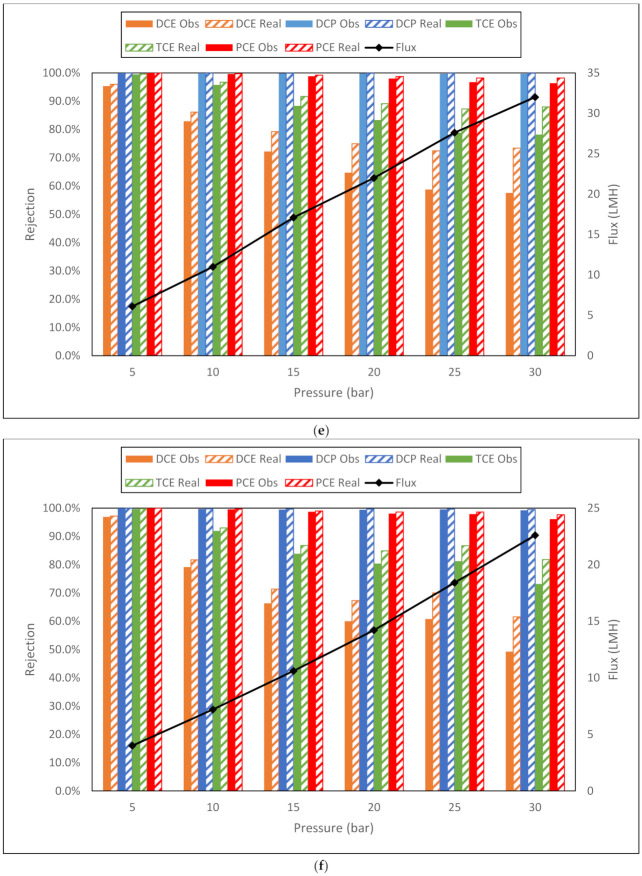

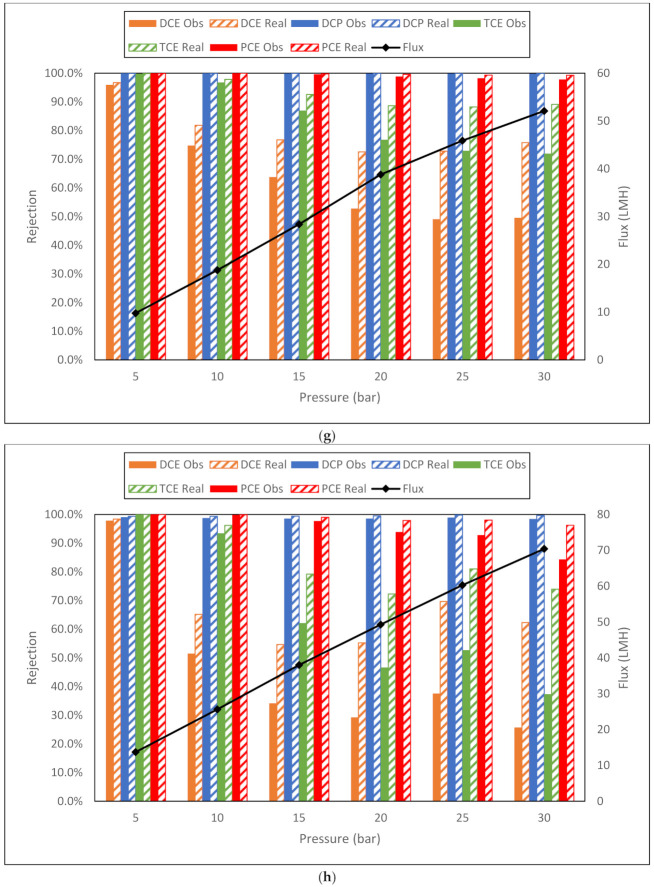

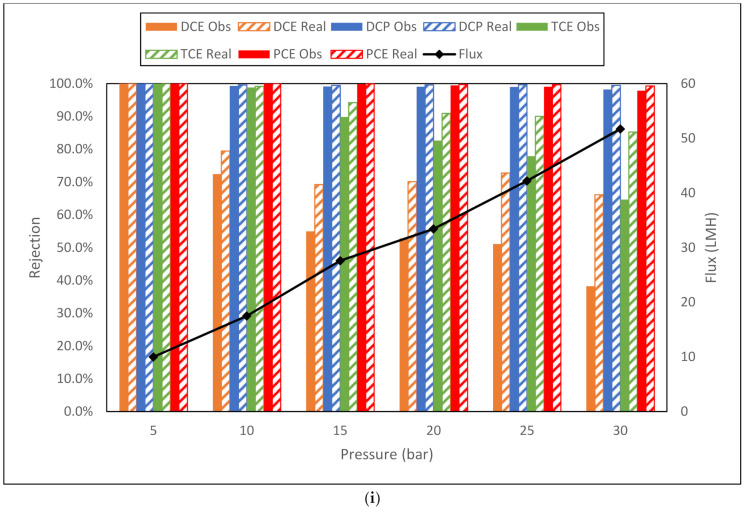
(**a**) Dow NF90 membrane flux (LMH) and observed and real rejection data for VOC solution using GC–MS HS analysis. (**b**) Dow N270 membrane flux (LMH) and observed and real rejection data for VOC solution using GC–MS HS analysis. (**c**) GE DK membrane flux (LMH) and observed and real rejection data for VOC solution using GC–MS HS analysis. (**d**) GE DL membrane flux (LMH) and observed and real rejection data for VOC solution using GC–MS HS analysis. (**e**) GE AK membrane flux (LMH) and observed and real rejection data for VOC solution using GC–MS HS analysis. (**f**) GE AG membrane flux (LMH) and observed and real rejection data for VOC solution using GC–MS HS analysis. (**g**) Dow BW30 membrane flux (LMH) and observed and real rejection data for VOC solution using GC–MS HS analysis. (**h**) Dow BW30LE membrane flux (LMH) and observed and real rejection data for VOC solution using GC–MS HS analysis. (**i**) Dow BW30XFR membrane flux (LMH) and observed and real rejection data for VOC solution using GC–MS HS analysis.

The TOC system at Swansea university was not equipped with the optional purgeable organic carbon (POC) analyzer. VOCs are easily purged from a sample via sparging, the TOC of the sample can be determined by the addition of the POC and non-purgeable organic carbon (NPOC). Despite the TOC method used not including a sparge of the sample, volatiles can be difficult to quantify through the subtraction method compared to the addition method, TOC = TC − IC and TOC = POC + NPOC, respectively. The analysis of volatile organic compounds (VOCs) in environmental water samples is usually performed by either headspace (HS) or purge and trap (P&T), with separation by gas chromatography (GC) and detection by mass spectrometry (MS). The P&T method uses a carrier gas remove volatiles from the solution and these are caught in an adsorbent trap. The trap is then heated which releases the volatiles into the GC-MS for subsequent analysis. This method provides excellent sensitivity as the total sample is extracted. However, in comparison to other methods, P&T methods are generally more complicated to operate and maintain. They can also suffer from a degree of water carry over, which may lead to a loss in sensitivity, a loss of peak shape and, in some cases, sample cross contamination. The obvious alternative is the HS method which uses a closed sample arrangement. In this case, the sample vial containing the total solution is heated (and agitated in some systems) in order to drive the volatiles out of the solution and into the headspace of the vial. When this is the case, an equilibrium forms between the volatile contained in the solution and the headspace. This equilibrium can be shifted by the addition of salt to the sample. After a specified time, a portion of the headspace is transferred onto the GC–MS via a valve with a sample loop. This technique is robust and experiences few carryover problems as less water is transferred to the GC–MS. The GC–MS HS system was installed at Swansea university after the pilot-scale trials, but during the laboratory trial. Therefore, a mixture of VOCs was produced. See Section 3.3.1. This VOC mixture underwent the same experimental pressures as the TOC analysis. The rejection results are shown in Figure 10a–i.

The membrane fluxes recorded with the VOC mixture feed show an interesting difference within the NF membranes tested and the RO membranes compared to the fluxes documented during the TCE TOC analysis tests. The GE NF90 behaved differently compared to all the other membranes—the flux on average, increased 11% when the mixture of VOC was filtered. All the other investigated NF membranes exhibited a decrease in flux—on average, 4%, 16% and 10% for NF270, DK and DL, respectively. The RO membranes suffered a far greater deterioration in flux—BW30 and BW30LE lost 31% of their permeate flux. Dow BW30XFR flux was down by 41% and 34% for the GE Osmonics AK membrane when compared to the TOC trials. The greatest drop in permeate flow rate was suffered by the AG membrane—a 74% loss. Upon examining the rejections from the GC–MS quantitation data, the GC–MS results generally agree with the TOC analysis—an increase in pressure decreases the solute retention. The reduction in rejection for the nanofiltration membranes was more severe than the equivalent TOC results for TCE—the lowest TCE rejection for TOC was between 21% and 47% for the four NF membranes. The equivalent GC–MS rejections observed were 0% for all four membranes at the highest operating pressure. This large discrepancy demonstrates the inaccuracies of using TOC as a method of substantiating the levels of VOCs in an aqueous sample. Concentration polarization is evident once again for the NF membranes. The largest difference of observed to real rejection was seen with DCP using NF270 at 30 bar, 8.8% to 97.1%, respectively—an 88.3% change. The NF membranes show good separation of TCE and PCE at 5 bar. DCE and DCP separation, however, would be considered poor across the pressure range. All the RO membranes demonstrate very good separation of PCE across the pressure range. TCE separation is good for observed rejection, and once concentration polarization is considered, the rejection increases to very good. Observed and real rejection both decrease as the pressure increases. DCE rejection remains very poor above 5 bar applied pressure for all RO membranes, particularly the low-energy BWRO Dow Filmtec BW30LE. In fact, the low-energy BW30LE performs worse compared to the standard RO membrane BW30 for all tests. Low-energy RO membranes are designed to operate at a significantly reduced operating pressure, whilst maintaining the separation capabilities and permeate flow rates of a standard RO operating at high pressure, this allows vastly reduced operating costs, making RO desalination much more appealing. This study has proven that this is not strictly true—there is a clear loss in rejection proficiency with an increase in permeate flow rate when comparing a standard RO to a low-energy RO at the same operating pressure. Previous and more recently published research [47,48] have suggested that solvent passage through an NF or RO membrane is due to the convection of the solvent to the membrane surface—adsorption of the VOCs through the membrane matrix followed by desorption from the membrane into the low concentration permeate. From the results, this study would confirm this theory. It has also been suggested that pressure has little to no effect on the rejection with this theory [47]. However, the experimental data presented in this study show this not to be the case. An increase in pressure increases the permeate flow rate, as a result the convection of solute towards the membrane surface increases, increasing concentration polarization. This increase in concentration polarization and permeate flow rate increases the rate of adsorption and desorption through the membrane matrix, respectively. Despite the continuous stirring within the cell, concentration polarization is made more likely due to the use of a frontal or dead-end filtration cell. A cross-flow filtration cell, where the feed is passed across the membrane surface rather than directly downwards onto surface, would reduce the possibility of concentration polarization occurring. The variations of adsorption kinetics have been attributed to the hydrophilicity or hydrophilicity of the membrane surface and the solvent molecule [48,49]. Solvents considered hydrophilic have a higher logarithm octanol–water partitioning coefficient, XLogP3 > 2.5, hydrophobic VOCs, XLogP3 < 2.5. The six highlighted VOCs throughout this paper are listed in Table 1, from most hydrophobic to hydrophilic. If the theory of hydrophilicity rejection is correct, then the rejection of the GC–MS results should be as follows (lowest to highest rejection) DCE < DCP < TCE < PCE. However, the rejection data from this study contradict this theory—DCP rejections are higher than TCE and PCE across the pressure range for all five RO membranes. DCP was not a VOC investigated by the previous researchers. The referenced study [48] also researched the rejection characteristics over a prolonged period of time, and it was determined that the concentration of VOCs in the permeate steadily increases until no separation occurs after 24 h. This would agree with the adsorption theory and the decrease in rejection at higher pressures observed in this study. An increase in pressure is simply speeding up the rate of adsorption and desorption that is experienced over a longer timeframe at a low pressure. All the membranes tested in this study were hydrophilic, having a contact angle <90 °C, the use of a membrane with a hydrophobic surface may present better selectivity of VOCs. Conversely, a hydrophobic membrane will experience a significant reduction in membrane flux, permeate flow rate, compared to the analogous hydrophilic membrane. Hence, a compromise between selectivity and permeate production rate must be balanced—a common situation to contemplate when selecting a suitable membrane for an application.

## 4. Conclusions

The results reported in this study demonstrate that an RO membrane has the potential to separate some VOCs that can be found in a contaminated groundwater source. The use of an extra fouling-resistant membrane, BWRO, from Dow Filmtec BW30XFR, produced promising results for the removal of all tested VOCs at 5 bar, with 100% observed rejection. The use of RO membranes for VOC removal is, however, an issue at high pressures and over a prolonged period of operation. Rejection proficiencies decline as the permeate flux increases, which in turn increases the convection of the VOCs to the membrane surface, causing concentration polarization at the membrane wall. An inevitable increase in concentration polarization results in a higher probability of adsorption of VOCs onto the membrane. The same principal explains the decline in VOC retention over time, due to adsorption of VOCs through the membrane matrix. The use a frontal filtration in the study compounds this problem. DCE was a compound that was inefficiently separated for all membranes tested at pressures above 5 bar. Hydrophobic VOCs generally appear to be more susceptible to adsorption than their counterparts, the hydrophilic VOCs. However, DCP would either discredit this correlation or is simply an anomaly to the rule. Further, in-depth study of VOCs is required to determine the validity of the suggested hypothesis.

## Figures and Tables

**Table 1:** Summary of physio-chemical properties of selected VOCs.

Compound	CAS #	Formula	MW (g/mol)	XLogP3
Vinyl Chloride	75-01-4	C2H3Cl	62.50	1.5
Cis-1,2-Dichloroethylene	156-59-2	C2H2CL2	96.94	1.9
2,2-Dichloropropane	594-20-7	C3H6Cl2	112.981	2.1
Trichloroethylene	79-01-6	C2HCl3	131.39	2.6
Toluene	108-88-3	C7H8	92.141	2.7
Tetrachloroethylene	127-18-4	C2Cl4	165.83	3.4

**Table 2:** GC–MS HS method settings.

Headspace Parameters	Agilent 7697A HS—12 vial		
**Temperature settings**			
Oven temperature	70 °C		
Loop temperature	85 °C		
Transfer line temperature	120 °C		
**Timing settings**			
Vial equilibration	10 min		
Injection duration	0.3 min		
GC cycle time	22 min		
**Vial settings**			
Vial size	20 mL		
Vial pressurization	15 psi		
Loop size	1.0 mL		
Extraction time	0.3 min		
Mode	Single extraction		
Transfer line flow	20 mL/min		
Transfer line	Agilent p/n 160-2535-5		
Line type	Fused silica, deactivated		
Line diameter	0.53 mm		
Vial and cap	20 mL, PTFE/silicone septa		
**GC Parameters**	**Agilent 6850 series II GC**		
**Inlet settings**			
Heater	On—150 °C		
Pressure	On—6.4 psi		
Total flow	On—42.6 mL/min		
Run time	6.8 min		
Gas saver	Off		
Split ratio	40:1		
Split flow	40 mL/min		
**Oven settings**			
Oven ramp	°C/min	Next °C	Hold
Initial		30	0.3 min
Ramp 1	5	55	0 min
Ramp 2	10	70	0 min
Total run time	15.8 min		
Equilibration time	0.5 min		
Oven Max temperature	260 °C		
Column	Agilent J & W HP-5		
Length	30 m		
Diameter	0.25 mm		
Film thickness	0.25 μm		
Mode	Constant flow		
Pressure	6.4 psi		
Nominal initial flow	1 mL/min		
Inlet	Front		
Outlet	MSD		
Outlet pressure	Vacuum		
**MSD Parameters**	**Agilent 5977A MSD**		
**MSD settings**			
Solvent delay	0.0 min		
Sim ions	4		
Quantitation ions	61, 77, 130, 166 M/Z		
Sim dwell	50 msec/ion		
Quad temperature	150		
Source temperature	230		
Transfer line temperature	250		
Gain factor	5		

**Table 3:** Characteristics and supporting information for the membranes used in this study.

a. Properties of NF membranes used in this study.									
**Membrane**		**DK**		**DL**		**NF90**		**NF270**	
Manufacturer		GE Osmonics		GE Osmonics		Dow Filmtec		Dow Filmtec	
Support material		Polysulfone		Polysulfone		Polysulfone		Polysulfone	
Surface material		TFC PA		TFC PA		PA		PA	
Maximum operating temperature		50 °C		50 °C		45 °C		45 °C	
Maximum operating pressure		41 bar		41 bar		41 bar		41 bar	
pH range		3–9		3–9		2–11		2–11	
Flux (GFD)/psi		22/100		28/220		46–60/130		72–98/130	
MWCO		~150–300		~150–300		~200–400		~200–400	
Contact angle		26.36 ± 0.48°		27.60 ± 0.38°		27.40 ± 0.89°		21.22 ± 0.88°	
b. Properties of RO membranes used in this study.									
**Membrane**	**AK**		**AG**		**BW30**		**BW30LE**		**BW30XFR**
Manufacturer	GE Osmonics		GE Osmonics		Dow Filmtec		Dow Filmtec		Dow Filmtec
Support material	Polysulfone		Polysulfone		Polysulfone		Polysulfone		Polysulfone
Surface material	TFC PA		TFC PA		PA		PA		PA
Maximum operatingtemperature	50 °C		50 °C		45 °C		45 °C		45 °C
Maximum operatingpressure	27 bar		41 bar		41 bar		41 bar		41 bar
pH range	4–11		4–11		2–11		2–11		2–11
Flux (GFD)/psi	26/115		26/225		26/225		37–46/225		28–33/225
MWCO	~0		~0		~100		~100		~100
Contact angle	70.21 ± 1.09°		72.21 ± 2.52°		55.86 ± 0.67°		67.58 ± 0.30°		56.61 ± 0.66°

**Table 4:** TOC data for dead-end filtration of the Bedfordshire site water.

1 g/L Toluene–Water Dead-End Filtration		
Sample Name	TOC (mg/L)	Rejection
Desal DK feed	508.6	
Desal DK 2.5 bar permeate	34.15	93.29%
Desal DK 5 bar permeate	108.5	78.67%
Desal DK 10 bar permeate	127.1	75.01%
Desal DK 20 bar permeate	154.2	69.68%

**Table 5:** TOC data for dead-end filtration of the Bedfordshire site water.

Company a Water Dead-End Filtration		
Sample Name	TOC (µg/L)	Rejection
Desal DK feed	3913	
Desal DK 2.5 bar permeate	316.4	91.91%
Desal DK 5 bar permeate	37.5	99.04%
Desal DK 10 bar permeate	0	100.00%
Desal DK 20 bar permeate	0.11	~100.00%

**Table 6:** GC–MS data of five main components detected for the 27th May samples.

Compound	Feed 1 (µg/L)	Feed 2 (µg/L)	Permeate 1 (µg/L)	Permeate 2 (µg/L)
Total	5476	5684	5662	5639
VC	6.6	6.5	7.5	7.3
DCE	1687	1758	1974	1749
DCP	177.4	188.6	212	180.1
TCE	3594	3720	3457	3691
PCE	7.9	7.6	8.1	7.7

**Table 7:** Swansea University TOC data for NF trial.

Date	Sample	Total Carbon (µg/L)	Inorganic Carbon (µg/L)	Total Organic Carbon (µg/L)	Average (µg/L)	Rejection
**13/05/2015**	Feed	89,796	82,635	7161	7598	57.19%
	Feed	90,388	82,353	8035		
	Permeate	64,208	60,934	3274	3253	
	Permeate	63,759	60,527	3232		
**27/05/2015**	Feed	84,059	76,947	7112	7314	70.30%
	Feed	83,069	75,553	7516		
	Permeate	65,731	63,566	2165	2172	
	Permeate	65,714	63,535	2179		
**09/06/2015**	Feed	80,894	74,534	6360	6360	78.30%
	Permeate	62,659	61,279	1380	1380	
	Feed	79,405	73,265	6140	6140	79.10%
	Permeate	59,334	58,051	1283	1283	
**23/06/2015**	Feed	75,762	70,445	5317	5317	71.96%
	Permeate	60,341	58,850	1491	1491	

**Table 8:** Nanofiltration membrane performance data.

Date	Inlet (bar)	Outlet (bar)	TMP (bar)	Recycle (l/s)	Permeate Flow (usgph)	Permeate Flow (l/h)	Flux (LMH)	Specific Flux (LMH/bar)
13th May	2.85	1.65	2.25	1.04	4.50	17.03	10.65	4.73
19th May	2.60	1.40	2.00	1.05	3.40	12.87	8.04	5.75
19th May	3.00	1.80	2.40	1.05	4.20	15.90	9.94	5.52
27th May	2.60	1.40	2.00	1.04	3.20	12.11	7.57	5.41
27th May	3.00	1.80	2.40	1.05	4.60	17.41	10.88	6.05
1st June	2.95	2.40	2.68	1.04	4.00	15.14	9.46	3.94
9th June	2.80	1.60	2.20	1.02	4.00	15.14	9.46	5.91
9th June	3.00	1.80	2.40	1.03	4.20	15.90	9.94	5.52
23rd June	2.50	1.30	1.90	1.02	3.00	11.36	7.10	5.46
23rd June	3.00	1.80	2.40	1.03	4.40	16.66	10.41	5.78
8th July	2.00	0.80	1.40	1.03	2.00	7.57	4.73	5.91

**Table 9:** GC–MS data of five main components detected for the 8th July samples.

Compound	Feed (µg/L)	Permeate (µg/L)	Rejection (%)
Total	3670.0	264.0	92.8
VC	6.1	1.8	70.3
DCE	1165.0	43.5	96.3
DCP	137.2	5.1	96.3
TCE	2351.7	175.8	92.5
PCE	7.1	0.6	91.7

**Table 10:** GC–MS data for five main components detected on 21th July.

Compound	Feed (µg/L)	Permeate (µg/L)
Total	6552	6613
VC	27.1	28.7
DCE	2636.9	2881.8
DCP	539.8	635.2
TCE	3340.8	3058.7
PCE	5.7	5.1

**Table 11:** RO membrane pilot-scale performance data.

Time	Inlet (bar)	Outlet (bar)	TMP (bar)	Recycle Flow (L/s)	Permeate Flow (L/h)	Flux (LMH)	Specific Flux (LMH/bar)
8th July	2.00	1.40	1.70	0.67	2398	2.12	1.51
21st July	3.00	1.60	2.30	1.03	3708	2.42	1.51
04th Aug	3.00	1.40	2.20	0.68	2441	1.97	1.41
18th Aug	3.00	1.40	2.20	*	*	1.51	1.08
1st Sept	2.60	0.70	1.65	*	*	0.30	0.43
1st Sept	3.00	1.50	2.25	*	*	1.67	1.11

* Unable to read totalizer.

**Table 12:** Laboratory separation assessment trials using nanofiltration and reverse osmosis membranes.

a. Dow NF90 membrane flux (LMH) and observed and real rejection data for NaCl, toluene and trichloroethylene from conductivity and TOC measurements.									
	**NaCl**			**Toluene**			**TCE**		
**Membrane**	**Flux**	**Obsd**	**Real**	**Flux**	**Obsd**	**Real**	**Flux**	**Obsd**	**Real**
NF90 5 bar	35.5	50.5%	69.2%	40.2	90.4%	95.8%	45.9	72.7%	88.1%
NF90 10 bar	80.4	58.6%	89.4%	85.8	70.5%	94.1%	86.1	42.6%	83.4%
NF90 15 bar	123	60.6%	95.9%	131.1	58.6%	96.3%	126.3	32.3%	88.7%
NF90 20 bar	172.2	52.4%	98.1%	179.5	49.7%	98.1%	163.9	32.6%	94.8%
NF90 25 bar	213.1	54.8%	99.3%	225.6	49.5%	99.3%	203.8	39.3%	98.4%
NF90 30 bar	236.5	32.1%	98.9%	275.6	51.7%	99.8%	241.1	32.7%	99.0%
b. Dow NF270 membrane flux (LMH) and observed and real rejection data for NaCl, toluene and trichloroethylene from conductivity and TOC measurements.									
	**NaCl**			**Toluene**			**TCE**		
**Membrane**	**Flux**	**Obsd**	**Real**	**Flux**	**Obsd**	**Real**	**Flux**	**Obsd**	**Real**
NF270 5 bar	45.9	48.6%	72.3%	54.5	83.5%	94.4%	47.8	53.9%	77.2%
NF270 10 bar	94.7	49.2%	88.8%	108.4	57.1%	93.6%	98.8	36.4%	83.7%
NF270 15 bar	137.8	50.4%	95.6%	157.7	45.6%	96.5%	155	29.2%	92.8%
NF270 20 bar	186	48.4%	98.3%	215.3	36.4%	98.6%	199.3	23.2%	96.2%
NF270 25 bar	230.4	38.1%	99.0%	259.4	36.0%	99.4%	258.4	21.5%	98.8%
NF270 30 bar	282.9	41.8%	99.7%	303.2	39.9%	99.8%	296.3	28.5%	99.7%
c. GE DK membrane flux (LMH) and observed and real rejection data for NaCl, toluene and trichloroethylene from conductivity and TOC measurements.									
	**NaCl**			**Toluene**			**TCE**		
**Membrane**	**Flux**	**Obsd**	**Real**	**Flux**	**Obsd**	**Real**	**Flux**	**Obsd**	**Real**
DK 5 bar	27.8	38.3%	53.5%	28.5	87.8%	93.1%	26.6	72.8%	82.9%
DK 10 bar	49.8	42.0%	68.6%	55.2	66.5%	87.1%	49.7	58.8%	81.2%
DK 15 bar	72.6	48.2%	82.4%	80.4	59.0%	89.6%	75.7	47.7%	83.1%
DK 20 bar	103.3	50.4%	91.0%	109.6	53.3%	92.8%	96.2	47.4%	88.4%
DK 25 bar	120.6	50.8%	93.8%	129.2	53.0%	95.2%	115.6	52.2%	93.4%
DK 30 bar	146.4	51.4%	96.5%	155	51.8%	97.1%	137.8	48.2%	95.2%
d. GE DL membrane flux (LMH) and observed and real rejection data for NaCl, toluene and trichloroethylene from conductivity and TOC measurements.									
	**NaCl**			**Toluene**			**TCE**		
**Membrane**	**Flux**	**Obsd**	**Real**	**Flux**	**Obsd**	**Real**	**Flux**	**Obsd**	**Real**
DL 5 bar	31.6	33.7%	50.6%	32.7	77.5%	87.7%	29.4	77.8%	87.0%
DL 10 bar	64.6	34.7%	69.0%	60.8	58.4%	84.4%	59.2	50.9%	79.4%
DL 15 bar	91.7	39.1%	83.1%	90	47.7%	87.0%	84.9	38.9%	80.7%
DL 20 bar	117.6	40.7%	90.3%	114.4	50.2%	92.7%	111.6	35.5%	86.7%
DL 25 bar	140.3	41.2%	94.0%	136.4	50.5%	95.5%	137.8	35.6%	92.2%
DL 30 bar	174.5	40.9%	97.1%	160.7	53.1%	97.6%	155	34.5%	94.3%
e. GE AK membrane flux (LMH) and observed and real rejection data for NaCl, toluene and trichloroethylene from conductivity and TOC measurements.									
	**NaCl**			**Toluene**			**TCE**		
**Membrane**	**Flux**	**Obsd**	**Real**	**Flux**	**Obsd**	**Real**	**Flux**	**Obsd**	**Real**
AK 5 bar	6.9	93.2%	94.1%	9.2	98.3%	98.6%	8.5	79.1%	82.1%
AK 10 bar	15.3	95.3%	96.6%	16.7	94.9%	96.4%	17.2	73.4%	80.2%
AK 15 bar	21.8	95.8%	97.3%	24.1	96.5%	97.9%	26.3	70.4%	81.0%
AK 20 bar	30.7	96.6%	98.3%	32.1	95.4%	97.7%	34.4	73.1%	85.4%
AK 25 bar	38.2	97.0%	98.7%	40.6	94.0%	97.5%	41.2	72.2%	86.6%
AK 30 bar	47.1	97.0%	98.9%	48.1	93.8%	97.8%	51.7	69.7%	87.9%
f. GE AG membrane flux (LMH) and observed and real rejection data for NaCl, toluene and trichloroethylene from conductivity and TOC measurements.									
	**NaCl**			**Toluene**			**TCE**		
**Membrane**	**Flux**	**Obsd**	**Real**	**Flux**	**Obsd**	**Real**	**Flux**	**Obsd**	**Real**
AG 5 bar	10.9	96.8%	97.5%	15.4	98.9%	99.3%	14.4	78.2%	83.2%
AG 10 bar	27.8	98.9%	99.4%	28.8	95.0%	97.3%	28.5	63.1%	76.3%
AG 15 bar	46.5	99.2%	99.7%	41.7	95.9%	98.3%	42.3	68.9%	85.0%
AG 20 bar	60.3	99.3%	99.8%	56.5	94.1%	98.2%	57.4	69.9%	89.3%
AG 25 bar	76.8	99.4%	99.9%	70.2	92.9%	98.4%	70.1	69.8%	91.6%
AG 30 bar	95.5	99.3%	99.9%	85.1	92.3%	98.8%	84	68.0%	93.2%
g. Dow BW30 membrane flux (LMH) and observed and real rejection data for NaCl, toluene and trichloroethylene from conductivity and TOC measurements.									
	**NaCl**			**Toluene**			**TCE**		
**Membrane**	**Flux**	**Obsd**	**Real**	**Flux**	**Obsd**	**Real**	**Flux**	**Obsd**	**Real**
BW30 5 bar	11.1	93.4%	94.7%	14	95.4%	96.6%	14.6	84.2%	88.1%
BW30 10 bar	24.4	94.9%	96.9%	27.7	96.7%	98.2%	27.3	79.0%	87.3%
BW30 15 bar	37.3	96.1%	98.2%	40.2	93.0%	97.0%	40.3	63.6%	81.1%
BW30 20 bar	52.6	96.6%	98.9%	53.5	92.0%	97.4%	54.5	52.2%	78.6%
BW30 25 bar	64.9	96.7%	99.2%	66	91.3%	97.9%	66	66.7%	89.7%
BW30 30 bar	77.5	96.9%	99.4%	78.9	90.7%	98.2%	79.6	65.1%	91.6%
h. Dow BW30LE membrane flux (LMH) and observed and real rejection data for NaCl, toluene and trichloroethylene from conductivity and TOC measurements.									
	**NaCl**			**Toluene**			**TCE**		
**Membrane**	**Flux**	**Obsd**	**Real**	**Flux**	**Obsd**	**Real**	**Flux**	**Obsd**	**Real**
BW30LE 5 bar	15.2	91.5%	93.8%	20.7	96.0%	97.5%	22.6	64.9%	75.3%
BW30LE 10 bar	35.9	94.3%	97.4%	41.1	91.2%	96.3%	43.2	47.6%	70.3%
BW30LE 15 bar	53.9	96.1%	98.8%	60.5	84.3%	95.4%	63.2	48.8%	79.5%
BW30LE 20 bar	77.5	96.1%	99.3%	79.8	83.1%	96.7%	87.2	48.5%	86.7%
BW30LE 25 bar	94.7	96.4%	99.5%	103.3	82.0%	97.8%	105.3	49.3%	91.0%
BW30LE 30 bar	112	96.6%	99.7%	112	77.0%	97.6%	127.4	65.2%	96.9%
i. Dow BW30XFR membrane flux (LMH) and observed and real rejection data for NaCl, toluene and trichloroethylene from conductivity and TOC measurements.									
	**NaCl**			**Toluene**			**TCE**		
**Membrane**	**Flux**	**Obsd**	**Real**	**Flux**	**Obsd**	**Real**	**Flux**	**Obsd**	**Real**
BW30XFR 5 bar	10	94.8%	95.8%	13.6	95.4%	96.5%	13	68.9%	74.7%
BW30XFR 10 bar	23	96.0%	97.6%	26.9	98.1%	98.9%	26.6	83.6%	90.2%
BW30XFR 15 bar	35.7	96.8%	98.5%	40.5	97.0%	98.8%	40.2	76.0%	88.5%
BW30XFR 20 bar	48.4	96.9%	98.9%	53.9	95.7%	98.7%	51.7	74.3%	90.1%
BW30XFR 25 bar	60.3	97.0%	99.2%	66	95.0%	98.8%	63.2	76.5%	93.0%
BW30XFR 30 bar	68.9	97.1%	99.4%	80.4	94.7%	99.1%	75.2	76.8%	94.6%

## Data Availability

The data presented in this study are available in article.
